# Residency Research Quality Outweighs Quantity in Predicting Sports Medicine Fellowship Placement

**DOI:** 10.1002/ars2.70027

**Published:** 2026-05-05

**Authors:** Carolina Gomez, Olivia Goldenberg, Sarah Haugh, Shreya M. Saraf, Mary K. Mulcahey

**Affiliations:** ^1^ Tulane University School of Medicine New Orleans Louisiana U.S.A.; ^2^ Loyola University Chicago Stritch School of Medicine Maywood Illinois U.S.A.; ^3^ Department of Orthopaedic Surgery & Rehabilitation Loyola University Medical Center Maywood Illinois U.S.A.

## Abstract

**Purpose:**

To determine whether academic productivity during residency correlates with the type of orthopaedic sports medicine fellowship program attended.

**Methods:**

All orthopaedic sports medicine fellowship programs accredited by the Accreditation Council for Graduate Medical Education were identified by cross‐referencing databases from the Arthroscopy Association of North America and the American Orthopaedic Society for Sports Medicine. An initial database search was conducted in September of 2024. Fellow and faculty names were collected from program websites or requested via email. Programs that did not list fellow or faculty names and did not provide them upon request were excluded. The research team followed up on the request for information if response was not received after 2 weeks. All programs that were listed in all 3 of the referenced databases and had fellow/faculty information publicly available or provided upon request were included in the study. Publication data were obtained using Scopus. Fellow academic productivity was assessed by H‐index and number of publications during residency. Faculty H‐index scores were averaged to represent each program's academic productivity.

**Results:**

Fifty‐seven programs met inclusion criteria. Programs were categorized as high or low academic productivity based on the median average faculty H‐index, and the threshold was determined to be 16.5. High‐academic‐productivity programs (n = 29) had a median faculty H‐index of 23.30 (mean: 27.46 ± 9.6), compared with 11.00 (mean: 10.89 ± 3.23) in low‐productivity programs (n = 28). A statistically significant but weak correlation was found between faculty H‐index and fellow H‐index (*P* = .045; Spearman's rho = 0.266). A weaker, nonsignificant correlation was observed between faculty H‐index and fellow publication count during residency (*P* = .075; Spearman's rho = 0.237). Subgroup analysis showed no significant differences in fellow H‐index or residency publication count between high‐ and low‐productivity programs (*P* = .060 and *P* = .090, respectively).

**Conclusions:**

Fellows in more academically productive fellowship programs show slightly higher H‐indices overall, yet the correlation is weak and productivity alone does not predict the type of fellowship program attended.

**Clinical Relevance:**

These findings offer insight into how research productivity during residency may play a role in determining the level of academic productivity of sports medicine fellowship programs and where residents match.

## Graphical abstract

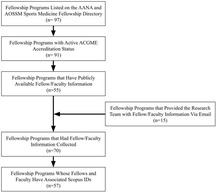

Most orthopaedic surgery residents pursue fellowship training after completing residency. Sports medicine fellowships are particularly popular among orthopaedic surgery residents, resulting in a highly competitive applicant pool for these programs.^1^ Applicants take several things into account when considering sports medicine fellowships in the United States including geographic location, academic institution, program director (PD), and research productivity of faculty. Many factors are thought to influence applicants’ chances of matching into orthopaedic sports medicine fellowships. In 2017, Baweja et al.^2^ showed that 37% of PDs listed the interview as the most important factor in ranking fellowship applicants. Letters of recommendation, an applicant's residency program, and publications were listed as the second through fourth most important factors, respectively. DeFroda et al.^3^ showed that there has been an overall increase in the number of publications among sports medicine fellowship applicants from 2010 to 2017.

A recent study by Clark et al.^4^ surveyed orthopaedic sports medicine fellowship PDs to determine the current research productivity of both fellows and faculty in Accreditation Council for Graduate Medical Education (ACGME)–accredited orthopaedic sports medicine fellowship programs in the United States. The authors found that applicants who desire to be academically productive during their fellowship year should consider programs with dedicated research assistants and/or programs that publish more than 25 manuscripts annually. However, only 33% of orthopaedic sports medicine fellowship programs participated, so the findings may not be broadly applicable.^4^ A clearer understanding of the academic productivity in sports medicine fellowships could help align a program's research goals with an applicant's research interests more effectively.

The Hirsch Index (H‐index) was developed to quantify scientific contribution. This measurement is an estimate of the importance, significance, and broad impact of a scientist's cumulative research contributions in a comparative, unbiased manner. The H‐index represents the number of studies that have been cited at least H times.^5^ For example, an author who has 5 published studies cited at least 5 times has an H‐index of 5. Previous studies have determined that higher H‐indices correlate with a higher academic rank for orthopaedic faculty.^6^, ^7^ Although H‐index is not a perfect measure, particularly for junior residents with few publications, it has been widely used in the orthopaedic literature to allow comparison across individuals and institutions. Haimowitz et al.^8^ investigated academic advancement of orthopaedic surgeons and found that orthopaedic subspecialty selection was independently associated with research productivity and academic rank. In 2021, Mayfield et al.^9^ concluded that most research is published by a relatively small number of top sports medicine fellowship programs and attendings in the United States, and the number of fellows or faculty does not significantly affect the quality or quantity of their research productivity. According to this study, the H‐index among faculty at a top 10 sports medicine program by research output was 24.13 vs 15.02 across all sports medicine faculty. Although there are studies documenting faculty research productivity in sports medicine fellowship programs, the relationship between resident research productivity and sports medicine fellowship faculty research productivity has yet to be studied. The purpose of this study was to determine whether academic productivity during residency correlates with the type of orthopaedic sports medicine fellowship program attended. The hypothesis was that fellows with high academic productivity during residency attend fellowship programs with high average faculty H‐index.

## METHODS

This was a retrospective study using publicly available data from program websites and fellowship directories. A current list of ACGME‐accredited orthopaedic sports medicine fellowship programs was obtained from the Arthroscopy Association of North America sports medicine fellowship directory and the American Orthopaedic Society for Sports Medicine database.^10^, ^11^ Program accreditation status was then confirmed using an ACGME accreditation report. Only programs with an active ACGME accreditation status were included in the study.

**FIGURE 1:**
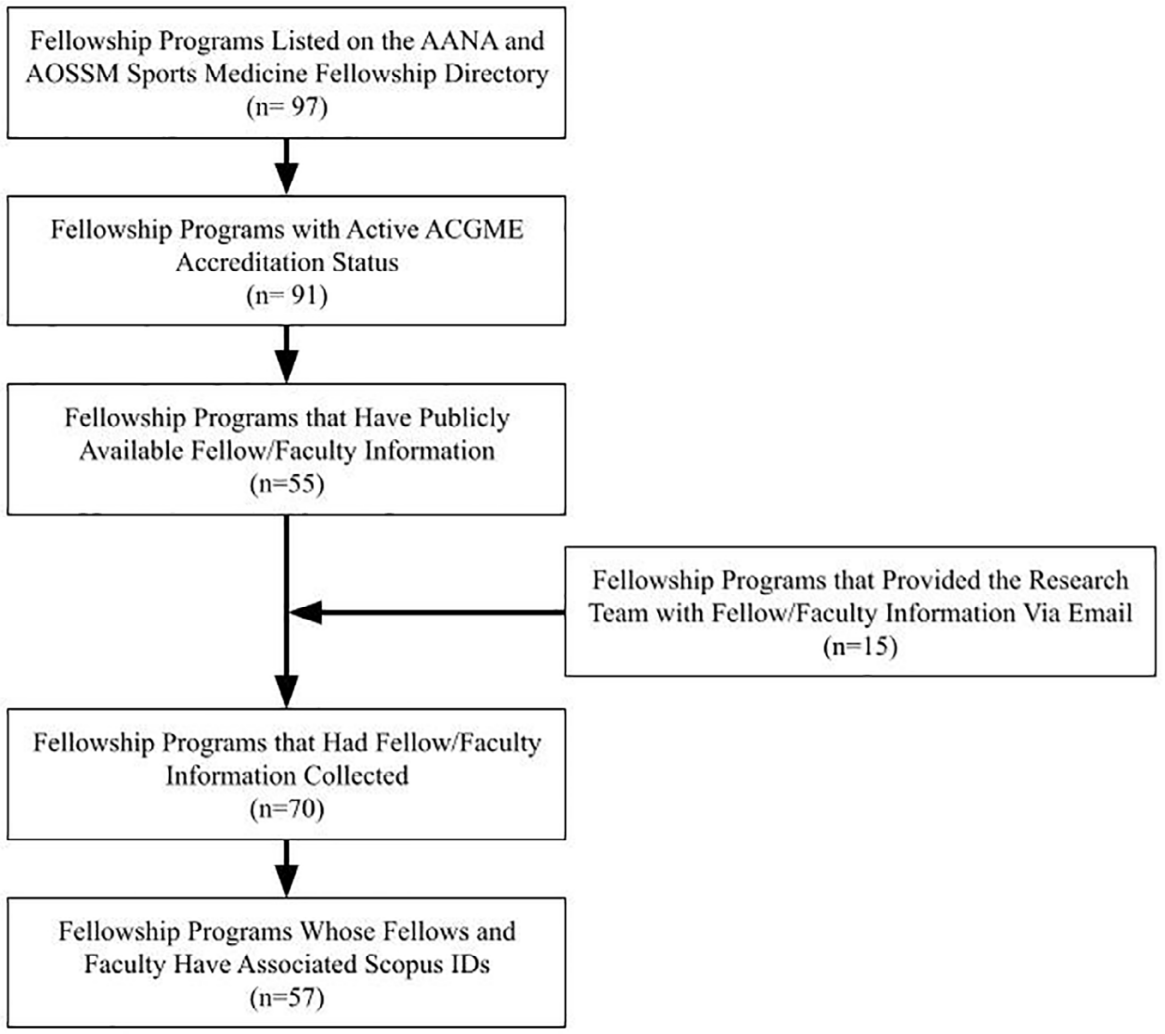
Flowchart for selection of programs included in the study. (AANA, Arthroscopy Association of North America; ACGME, Accreditation Council for Graduate Medical Education; AOSSM, American Orthopaedic Society for Sports Medicine.)

Data extraction was performed by 3 co‐authors (C.G., O.G., S.H.) experienced with bibliometrics and database querying. The names of the current fellows in each program were then obtained from the program's individual website along with residency programs attended, if available. A list of faculty members was obtained from each program's website along with the number of fellows in the program, number of faculty in the program, and the program's geographic location. If a program did not list fellows or faculty on the website, the fellowship coordinator was contacted via email and fellow/faculty information was requested. Programs that did not list fellow/faculty names on their website and did not provide this information via email were excluded. The selection process for programs included is shown in Figure 1.

The programs were categorized as having either high or low academic productivity based on their combined faculty H‐index. Programs with a combined faculty H‐index above the average for all orthopaedic sports medicine fellowship programs were classified as high academic productivity, whereas those below the average were classified as low academic productivity. Programs were then further divided into quartiles based on academic productivity as determined by the combined faculty H‐index.

### Data Extraction

Data extraction was performed by 3 co‐authors (C.G., O.G., S.H.). Scopus was used to identify each orthopaedic sports medicine fellow, their Scopus ID, and H‐index (residency affiliation was used to positively identify each fellow, if available). If there were multiple Scopus IDs identified for a single fellow, a manual count of publications and H‐index was conducted and the H‐index was calculated. To assess research productivity of each fellow during their residency, Scopus was used to collect the number of publications from the fellow's first year of residency to 1 year following the completion of their residency program. The H‐index was used as a measure of quality of research during residency, whereas the number of publications during residency was used to measure quantity. Scopus was then used to collect the H‐index and number of publications for every faculty member at each institution. The mean H‐index for the faculty members in each program was to determine the fellowship program's academic productivity. For statistical analysis, the fellowship programs were divided into 2 groups: either high‐academic‐productivity programs (average faculty H‐index >16.5) or low‐academic‐productivity programs (average faculty H‐index ≤ 16.5).

### Statistical Analysis

Statistical analysis was completed using SPSS, version 29.0 (IBM, Armonk, NY). The Shapiro‐Wilks test was used to determine the normality of the data and guide statistical analysis. Descriptive statistics including median, mean with standard deviation, minimum, and maximum were reported for the data in this study. Correlation analysis was performed for continuous data using Spearman's rho due to the nonparametric distribution of the data. The Mann‐Whitney U test was used to compare differences in fellow H‐index and fellow academic productivity during residency between the high‐academic‐productivity programs and the low‐academic‐productivity programs. The Kruskal‐Wallis test was used to identify a difference among the varying program academic quartiles for fellow H‐index and fellow number or publications during residency. Significance values were set at *P* < .05 for all analyses in this study.

## RESULTS

### Program Demographics

The median combined faculty H‐index for the high‐academic‐productivity programs (n = 29) was 23.30 (mean: 27.46 ± 9.6; range: 16.80‐54.50) compared with 11.00 (mean: 10.89 ± 3.23; range: 4.80‐16.50) for the low‐academic‐productivity programs (n = 28). The fellowship programs were then further divided into quartiles. The median combined faculty H‐index for Q1 programs (n = 15) was 32.80 (mean: 34.16 ± 8.96; range: 23.30‐54.50), compared with 8.25 (mean: 8.20 ± 1.85; range: 4.80‐10.80) for Q4 programs (n = 14). The average median, mean, standard deviation, minimum, and maximum values for the fellows in these various groups are noted in Table 1.

**TABLE 1:** Fellow Demographics for Various Groups

Program Group	Fellow Measure	Median	Mean	Standard Deviation	Min	Max
High‐academic‐productivity (n = 29)						
	Average H‐index	4.50	5.90	±4.59	1.00	19.50
	Average number of publications during residency	13.30	14.68	±14.81	1.50	54.80
Low‐academic‐productivity (n = 28)						
	Average H‐index	2.85	3.61	±2.92	0	13.00
	Average number of publications during residency	5.50	7.68	±6.69	1.00	25.00
Q1 (n = 15)						
	Average H‐index	5.70	6.49	±3.95	1.20	13.60
	Average number of publications during residency	13.70	17.38	±15.58	3.00	54.80
Q2 (n = 14)						
	Average H‐index	3.15	5.26	±5.26	1.00	19.50
	Average number of publications during residency	4.40	11.78	±13.92	1.50	48.50
Q3 (n = 14)						
	Average H‐index	2.50	3.66	±3.61	0	13.00
	Average number of publications during residency	4.50	7.91	±8.21	1.00	25.00
Q4 (n = 14)						
	Average H‐index	3.00	3.55	±2.15	1.00	8.0
	Average number of publications during residency	6.40	7.45	±5.05	2.30	18.00

### Academic Productivity During Residency and Fellowship Program Attended

**FIGURE 2:**
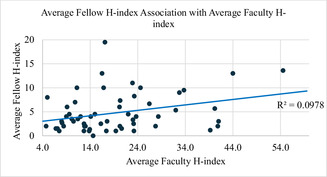
Average fellow H‐index association with average faculty H‐index.

**FIGURE 3:**
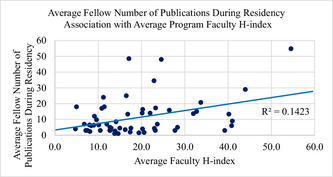
Average fellow number of publications during residency association with average program faculty H‐index.

There was a significant but weak correlation between the average faculty H‐index and fellow H‐index (*P* = .045; Spearman's rho = 0.266) (Figure 2). Further analysis also revealed a weak correlation that was not statically significant between faculty H‐index and fellow academic productivity during residency (*P* = .075; Spearman's rho = 0.237) (Figure 3). Quality of research had a stronger correlation to the type of fellowship program attended when compared with quantity of research.

Subgroup analysis comparing fellow H‐index and fellow academic productivity during residency across the high‐academic‐productivity programs and the low‐academic‐productivity programs revealed that there was no significant difference in the fellow H‐index or fellow academic productivity during residency across the 2 groups (*P* = .060, *P* = .090, respectively). Additional subgroup analysis comparing fellow H‐index and fellow academic productivity during residency across the 4 quartile fellowship program groups showed that neither the fellow H‐index nor fellow academic productivity during residency differed significantly across the various program quartiles as reported in Table 2 (*P* = .128, *P* = .106, respectively).

**TABLE 2:** Subgroup Analysis Comparing Fellow H‐Index and Fellow Number of Publications During Residency

	Fellow H‐Index (Median)	*P* Value	Fellow # of Publications During Residency (Median)	*P* Value
Academic productivity ranking		.060*		.090*
High‐academic‐productivity	4.50		13.30	
Low‐academic‐productivity	2.85		5.50	
Quartile		.128†		.106†
1st	5.70		13.70	
2nd	3.15		4.40	
3rd	2.50		4.50	
4th	3.0		6.40	

* Mann‐Whitney U test (nonparametric).

† Kruskal‐Wallis test (nonparametric).

## DISCUSSION

We found that orthopaedic sports medicine fellows with higher H‐indices generally attend fellowship programs that are more academically productive. However, the additional analyses conducted suggest that the quality of research may be a more predictive measure of the type of fellowship program residents attend compared with the quantity of research performed.

There is currently wide variation in what sports medicine fellowship PDs consider most important. A study by Baweja et al.^2^ surveyed sports medicine fellowship PDs and determined that they consider research productivity during residency to be important contribution to an applicant's application. However, other factors such as the interview, letters of recommendation, and residency program are generally more important to PDs than the research productivity during residency. Although research was not ranked first overall in the study by Baweja et al.,^2^ a few programs listed research as the most important factor in ranking fellowship applicants.

Other studies that have analyzed the importance placed on research productivity for other orthopaedic surgery subspecialties have reported similar results. A study by Johnson et al.^12^ surveyed pediatric orthopedic fellowship PDs and most of the programs reported that other factors such as the applicants interview and letters of recommendation were more important to most PDs and only 10% of surveyed PDs reporting that research experience was the most important factor. Another study by Sandhu et al.^13^ also reported similar findings when surveying orthopaedic trauma fellowship PDs and asking which factors they deem most important when ranking applicants. Our study found that research productivity during residency does not have a direct correlation to fellowship program academic productivity; this is likely due to the PDs' consideration of other application components to be more important when ranking applicants. However, many of these studies still report a small percentage of highly academic programs that still prioritize an applicant's research productivity over other factors, which may explain why orthopaedic sports medicine fellows with higher H‐indices generally attend fellowship programs that are more academically productive.

This study provides insight to residents as to how research productivity during residency correlates with fellowship program academic productivity, which may help in selecting fellowship programs to interview at and rank, if research productivity is a priority. Future studies should elaborate on these results by surveying sports medicine fellowship PDs to determine a minimum level of residency research productivity necessary to be considered a qualified applicant for fellowship programs with varying levels of academic productivity. Additionally, researchers should consider collecting PD opinions on quality versus quantity of research and if one is considered to be more important when ranking applicants.

### Limitations

There are several limitations to this study. First, the H‐index was used as a measure of academic productivity. This is a widely used measure; however, there is some concern in the literature about the limitations that accompany the use of the H‐index as a measure of academic productivity.^14^ Second, several programs were excluded due to current fellow names or current faculty names not being publicly available. This decreased the sample size and did not allow for complete analysis of all current ACGME‐accredited orthopaedic sports medicine fellowship programs. Additionally, several H‐indices were self‐calculated to account for various Scopus IDs associated with the same fellow or faculty member. Another limitation was that the window for data extraction may include some research productivity from medical school. This may also be subject to selection bias, as inclusion was limited to orthopaedic sports medicine fellowship programs listed in all 3 databases (ACGME, Arthroscopy Association of North America, and American Orthopaedic Society for Sports Medicine) with available faculty or fellow information. Exclusion of programs lacking this information may have resulted in an overrepresentation of programs with greater academic visibility and infrastructure, limiting generalizability to all sports medicine fellowships. Lastly, Scopus was the only database used to retrieve publication metrics. This limits the present study's ability to ensure that all publications by individual authors were included in the analysis.

## CONCLUSIONS

Fellows in more academically productive fellowship programs show slightly higher H‐indices overall, yet the correlation is weak and productivity alone does not predict the type of fellowship program attended.

## DISCLOSURES

The authors (C.G., O.G., S.H., S.M.S., M.K.M.) declare that they have no known competing financial interests or personal relationships that could have appeared to influence the work reported in this article.
